# Expression of IL-4 and IL-12 in the aqueous humor of patients with chronic primary angle-closure glaucoma

**DOI:** 10.3389/fmed.2024.1323829

**Published:** 2024-04-08

**Authors:** Qi Feng, Jiang Shen, Li Zhang, Qiong Wang, Surong Luo, Man Luo, Wei Chen

**Affiliations:** Department of Ophthalmology, Shaoxing People's Hospital, Zhejiang University, Shaoxing, China

**Keywords:** aqueous humor, cytokine, retinal ganglion cells, primary angle-closure glaucoma, retinal nerve fiber layer

## Abstract

**Introduction:**

Angle-closure glaucoma is a common type of glaucoma in Asian populations. However, the role of inflammatory cytokines in angle-closure glaucoma is yet to be elucidated. Therefore, this study aimed to examine the expression of interleukin-4 (IL-4) and IL-12 in the aqueous humor of patients with chronic primary angle-closure glaucoma (CPACG) and elucidate the correlations between IL-4 and IL-12 concentrations in the aqueous humor, the degree of visual field defects, and retinal nerve fiber layer (RNFL) thickness in patients with CPACG.

**Methods:**

Aqueous humor samples were obtained from 31 patients diagnosed with CPACG at the Shaoxing People’s Hospital between April 2022 and March 2023 and from 30 individuals with cataract (control). Based on the degree of the mean defect (MD), patients were divided into three groups: group A (MD ≤ −6dB, n= 10), group B (−6dB< MD< −12dB, n= 9), and group C (MD ≥ − 12dB, n= 12). RNFL thickness was measured using an optical coherence tomograph, and the concentrations of IL-4 and IL-12 in the aqueous humor were measured using Luminex technology.

**Results:**

Aqueous humor concentration of IL-4 was significantly higher (*p* = 0.036) in the CPACG group than in the cataract group. However, there was no significant difference (*p* > 0.05) in IL- 12 concentration between the two groups. Additionally, there were no significant differences (*p* > 0.05) in IL-4 and IL-12 levels among patients with varying degrees of visual field defects (groups A, B, and C). Spearman’s correlation analysis showed that IL-4 and IL-12 concentrations were not correlated (*p* > 0.05) with RNFL thickness around the optic disc and the degree of visual field defects.

**Discussion:**

Conclusively, IL-4 may play an important role in the pathogenesis of CPACG. Given that IL-4 and IL-12 concentrations in the aqueous humor were not significantly correlated with RNFL thickness and the degree of visual field defects, the increase in IL-4 and IL-12 expression may not induce apoptosis and loss of retinal ganglion cells or affect RNFL thickness as well as the degree of visual field defects.

## 1 Introduction

As a group of ocular conditions, glaucoma causes progressive damage to the optic nerve with corresponding damage to the visual field. In terms of irreversible blindness, it is the second most common cause. Glaucoma is associated with apoptosis of retinal ganglion cells and their axons, which results in a decrease in the thickness of the retinal nerve fiber layer (RNFL). As a result of all these pathological changes, peripheral visual field defects progress, resulting in blindness (1). Primary angle-closure glaucoma (PACG) and primary open-angle glaucoma (POAG) are the two types of primary glaucoma. Approximately 3.54% of the population aged 40–80 years has glaucoma, with POAG being the most prevalent in Africa (4.2%) and PACG being the most common in Asia (1.1%). Statistically, 111.8 million people worldwide is predicted to suffer from glaucoma by 2040 (2). Recently, glaucoma has become a serious public health concern owing to an increase in the aging population and its higher prevalence among older individuals.

Glaucoma is strongly influenced by elevated intraocular pressure during its development and progression (3). A variety of factors play an important role in the occurrence and development of glaucoma, including immune response, inflammation, hypoxia, oxidative stress, and others. Recently, the role of immune factors in glaucoma pathogenesis has attracted research interest. In ocular diseases, the aqueous humor can accurately reflect the internal environment of the eye. Accordingly, the understanding of the pathophysiology of glaucoma can be improved by assessing the levels of cytokines in the aqueous humor. It has been reported that ocular diseases such as glaucoma, uveitis, macular degeneration, and others cause cytokine changes in the aqueous humor (4–7). Specifically, several inflammatory factors are upregulated in the aqueous humor of patients with PACG and POAG, with a more prominent increase observed in patients with POAG (8).

Interleukin-4 (IL-4) is a major cytokine involved in immune responses and is mainly secreted by T lymphocytes. As the most potent natural killer (NK) cell activator, IL-12 stimulates the proliferation and activation of NK cells, as well as induces NK cells and other immune cells crucial for the adaptive immune response (9). IL-4 and IL-12 play important roles in cellular immune responses. Although some preliminary research on the role of cytokines in glaucoma has been performed, investigating the levels of IL-4 and IL-12 in the aqueous humor of patients with glaucoma is necessary to improve our understanding of the pathogenesis of glaucoma and facilitate research on potential therapeutics.

Previous studies have mainly focused on the concentrations of IL-4 and IL-12 in the aqueous humor of patients with POAG, and information on the levels of both cytokines in the aqueous humor of patients with chronic primary angle-closure glaucoma (CPACG) is limited. Moreover, the correlations between IL-4 and IL-12 levels in the aqueous humor of patients with CPACG, peripapillary RNFL thickness, and the degree of visual field defect are unknown. Therefore, this study aimed to elucidate the concentrations of IL-4 and IL-12 in the aqueous humor of patients with CPACG and investigate the correlations between IL-4 and IL-12 levels in the aqueous humor, peripapillary RNFL thickness, and the degree of visual field defect. We believe that comprehensive understanding of the role of IL-4 and IL-12 in the occurrence and progression of CPACG would facilitate research on effective treatment strategies.

## 2 Materials and methods

### 2.1 Study subjects

Thirty-one patients diagnosed with CPACG at Shaoxing People’s Hospital between April 2022 and March 2023 were included in the observation group. Based on the degree of the mean defect (MD), patients in the observation group were divided into three categories: groups A (MD ≤ −6 dB, mild optic nerve damage, n = 10), B (−6 dB < MD < −12 dB, moderate damage to the optic nerve, *n* = 9), and C (MD ≥ −12 dB, severe damage to the optic nerve, *n* = 12). Healthy Chinese individuals (*n* = 30) with cataracts were used as the control group and were matched for age. In this study, the Ethics Committee of Shaoxing People’s Hospital approved the experiment (No.: 2021-K-Y-55-01), and duly signed informed consent was obtained from the patients.

Patients with CPACG were treated with drugs to reduce intraocular pressure to ≤20–30 mmHg (1 mmHg = 0.133 kPa). Notably, visual field examination showed that patients with CPACG had different degrees of visual field defects. Individuals in the control group were diagnosed with uncomplicated age-related cataracts and had no evidence of glaucomatous optic neuropathy or other ocular diseases. Patients with severe heart disease, hepatic insufficiency, renal insufficiency, pre-existing ocular disease (such as diabetic retinopathy, retinal vein occlusion, retinal artery occlusion, and age-related macular degeneration) were excluded from the study.

Basic information about the participants, such as age, sex, and concomitant diseases, was obtained. All participants underwent comprehensive ophthalmic examinations, including visual acuity, slit-lamp anterior segment, intraocular pressure, and fundus examinations. Additionally, patients with CPACG were subjected to further visual field and optical coherence tomography examinations to determine the peripapillary RNFL thickness. An experimental group of patients underwent goniosynechialysis as well as phacoemulsification with microincisions and intraocular lens implantation, Patients in the experimental group underwent goniosynechialysis combined with microincision phacoemulsification and intraocular lens implantation, whereas patients in the control group only underwent microincision phacoemulsification and intraocular lens implantation.

### 2.2 Sampling of aqueous humor

Aqueous humor samples were obtained from all patients under sterile conditions before the surgery. Briefly, 100 μL of aqueous humor was extracted via anterior chamber puncture using a 25-gauge needle syringe to ensure that the intraocular tissue was not touched. All aqueous humor samples were immediately frozen and stored at −80°C waiting for analysis. Multiplex Luminex technology (Luminex, Austin, TX, United States) was used to measure IL-4 and IL-12 concentrations in aqueous humor. Luminex technology is a multifunctional liquid phase analysis platform based on colored microspheres, laser technology, applied fluid science, and high-speed digital signal processing technology.

### 2.3 Statistical analysis

All statistical analyses were performed using SPSS version 25.0 software (IBM Corp, Armonk, NY, United States). The count data are expressed as frequency, while measurement data are expressed as mean ± standard deviation. Significant difference between normally distributed data was determined using one-way analysis of variance, while non-normal data were compared using the Wilcoxon rank-sum test. Spearman’s correlation analysis was performed to determine the relationship between cytokine expression levels, the degree of visual defect, and RNFL thickness. *p* ≤ 0.05 was set as the level of statistical significance.

## 3 Results

### 3.1 Patient demographics and clinical characteristics

In total, 61 participants (32 males and 29 females), including 31 individuals with CPACG (experimental group) and 30 patients with age-related cataracts (control group), were enrolled in this study. The mean ages of individuals in the CPACG and cataract groups were 67.81 ± 6.99 (range 51–76) and 70.30 ± 7.39 (range 57–88) years, respectively (Table 1). No significant differences (*p* > 0.05) in sex, age, or general condition between the CPACG and the cataract groups were observed (Table 1).

**Table 1:** Demographics and clinical characteristics of CPACG and cataract.

Characteristics	CPACG (*n* = 31)	cataract (*n* = 30)	*X*^2^/Z	*p*
Sex			1.971	0.160
Female	19 (61.29%)	13 (43.33%)		
Male	12 (38.71%)	17 (56.67%)		
Age (Mean ± SD)	67.81 ± 6.99	70.3 ± 7.39	−1.084	0.278
Hypertension	11 (35.48%)	14 (46.67)	0.788	0.375
Diabetes	6 (19.35%)	1 (3.33%)	2.437	0.119
Other basic diseases*	6 (19.35%)	5 (16.67%)	0.075	0.785

^*^Other basic diseases include hepatic cysts, thyroid nodules, gout, prostatic hyperplasia, pancreatic cyst, hepatitis B, gallbladder stones, lung cancer, essential thrombocythemia, and cerebral infarction.

### 3.2 Aqueous cytokines analysis

Aqueous humor concentration of IL-4 was significantly higher (*p* < 0.05) in the CPACG group (10.11 ± 16.96 pg./mL) than in the control group (Table 2). However, there was no significant difference (*p* > 0.05) in IL-12 concentration between the two groups. Additionally, there were no significant differences (*p* > 0.05) in IL-4 and IL-12 levels among patients with varying degrees of visual field defect (groups A, B, and C). Specifically, the mean concentrations of IL-4 were 9.1 ± 15.28, 11.01 ± 117.13, and 10.27 ± 19.45 pg./mL in groups A, B, and C, respectively, whereas the concentrations of IL-12 were 12.74 ± 3.86, 12.59 ± 3.65, and 11.05 ± 4.12 pg./mL in groups A, B, and C, respectively (Table 3).

**Table 2:** Concentrations of cytokines in the aqueous humor of patients with CPACG and cataract.

	CPACG (*n* = 31)	Cataract (*n* = 30)	*Z*	*p*
IL-4 (pg/mL)	10.11 ± 16.96	3.00 ± 1.82	−2.101	0.036
IL-12 (pg/mL)	12.04 ± 3.86	13.23 ± 4.75	−1.212	0.225

**Table 3:** Concentrations of cytokines in the aqueous humor of patients in Groups A, B, and C.

	A	B	C	H	*p*
IL-4 (pg/mL)	9.1 ± 15.28	11.01 ± 17.13	10.27 ± 19.45	0.005	0.997
IL-12 (pg/mL)	12.74 ± 3.86	12.59 ± 3.65	11.05 ± 4.12	1.380	0.501

A Spearman’s analysis was performed to determine the correlation between IL-4 and IL-12 expression levels and RNFL thickness. There was no significant correlation (*p* > 0.05) between cytokine expression levels and RNFL thickness (Table 4).

**Table 4:** Correlation of IL-4 and IL-12 expression levels with RNFL thickness.

Variable	Mean ± SD	r	*p*
IL-4 (pg/mL)	10.11 ± 16.96	0.194	0.295
IL-12 (pg/mL)	12.04 ± 3.86	0.090	0.630

## 4 Discussion

Recently, a growing body of research is examining the role immunological factors play in glaucoma. Becke (10) was the first to detect plasma cells and immunoglobulins in glaucomatous trabecular biopsies, suggesting changes in humoral immunity. Subsequently, several studies reported abnormalities in serum cytokines, antibodies, and complement systems, and found that cytokine-mediated immune and inflammatory responses play an important role in the process of glaucomatous optic neuropathy. Notably, the aqueous humor of patients with glaucoma has higher concentrations of cytokines, such as IL-4, IL-6, IL-8, IL-10, vascular endothelial growth factor, and interferon-α, compared to healthy individuals (11, 12). Yin (13) found that the levels of tumor necrosis factor-alpha (TNF-α) and transforming growth factor -β2 were significantly higher in the aqueous humor of patients with POAG than in those of patients with cataract. Similarly, Kokubun (14) reported that the aqueous humor of patients with POAG and neovascular glaucoma (NVG) had a higher level of IL-8, IP-10, and MCP-1 than those of patients with cataract. However, POAG patients had lower levels of IL-12 in their aqueous humor than cataract and NVG patients. Although several studies have been performed on the levels of cytokines in the aqueous humor of patients with different types of glaucoma (1), such as POAG and NVG, there are few studies on the cytokine levels in patients with CPACG and PACG in the aqueous humor (11, 15).

IL-4 is produced by T-helper 2 (Th2) cells and participates in humoral immune responses (16). IL-12 is produced by activated antigen-presenting cells, including dendritic cells and macrophages, and is a potent inducer of interferon-γ production by T cells and NK cells (17). Currently, relatively few studies have investigated the levels of IL-4 and IL-12 in the aqueous humor of Chinese individuals, while only two studies have reported the serum levels of IL-4 and IL-12 in patients with POAG (18, 19). Both studies showed that patients with POAG had significantly higher serum IL-4 levels and significantly lower IL-12 levels than healthy individuals. IL-4 is presumed to have a damaging effect on optic nerve cells, whereas IL-12 has a protective effect. However, we think that the detection of inflammatory factors in serum is affected by multiple systemic factors and cannot simply represent the inflammatory response of the eye. Chono et al. found that the aqueous humor of patients with POAG had higher IL-4 and IL-12 levels than that of individuals in the control group (20). Similarly, Chau found that the POAG group had higher IL-12 concentrations than the control group, while the PACG group and control group did not show any significant differences (8).

In the present study, the aqueous humor of patients with CPACG had significantly higher expression levels of IL-4 than those of patients with cataracts, suggesting that IL-4 may be involved in the pathogenesis of CPACG. However, there was no significant difference in IL-12 levels between the two groups, consistent with the findings of Chau (8). Given that patients with POAG were not included in this study, we cannot conclude whether there is a significant difference in aqueous humor IL-12 levels between patients with POAG and PACG. Additionally, patients with CPACG were divided into mild, moderate, and severe groups according to the degree of visual field defect, and the concentrations of IL-4 and IL-12 in the aqueous humors of the three groups were analyzed. Notably, no significant differences were observed in the expression levels of either cytokine among the three groups. Moreover, Spearman’s analysis showed that the levels of IL-4 and IL-12 in the aqueous humor were not significantly correlated with RNFL thickness or the degree of visual field defects. Based on these results, we inferred that the increase in IL-4 and IL-12 concentrations may not induce apoptosis and loss of retinal ganglion cells (RGC) or affect RNFL thickness or the degree of visual field defects.

To the best of our knowledge, this is the first study to evaluate IL-4 and IL-12 levels in the aqueous humor of Chinese patients with CPACG. However, this study has certain limitations. For example, the use of anti-glaucoma medications may affect cytokine levels in the aqueous humor. Therefore, further studies should consider the effects of medications on cytokine levels in the aqueous humor. Additionally, the sample size of this study was small, which may limit the ability to draw definitive conclusions.

## 5 Conclusion

We reported, for the first time, that IL-4 level was significantly upregulated in the aqueous humor of patients with CPACG, suggesting an important role in the CPACG pathogenesis. IL-12 may have no significant effect on the pathogenesis of CPACG. Additionally, IL-4 and IL-12 levels in the aqueous humor were not significantly correlated with RNFL thickness or the degree of visual field defects. Our results suggest that the increase in IL-4 and IL-12 concentrations may not induce apoptosis and loss of RGC or affect RNFL thickness or the degree of visual field defects. However, further studies involving a large sample size are necessary to elucidate the role and mechanisms of immune and inflammatory factors in the development of CPACG.

## Data availability statement

The raw data supporting the conclusions of this article will be made available by the authors, without undue reservation.

## Ethics statement

The studies involving humans were approved by the Ethics Committee of Shaoxing People’s Hospital (No.: 2021-K-Y-55-01). The studies were conducted in accordance with the local legislation and institutional requirements. The participants provided their written informed consent to participate in this study.

## Author contributions

QF: Conceptualization, Writing – original draft, Software. JS: Investigation, Writing – original draft, Data curation. LZ: Writing – review & editing, Validation. QW: Investigation, Writing – original draft, Data curation. SL: Investigation, Writing – original draft, Data curation. ML: Data curation, Writing – original draft, Resources. WC: Methodology, Project administration, Writing – review & editing.
